# Interlaced zone plate optics for hard X-ray imaging in the 10 nm range

**DOI:** 10.1038/srep43624

**Published:** 2017-03-08

**Authors:** Istvan Mohacsi, Ismo Vartiainen, Benedikt Rösner, Manuel Guizar-Sicairos, Vitaliy A. Guzenko, Ian McNulty, Robert Winarski, Martin V. Holt, Christian David

**Affiliations:** 1 Paul Scherrer Institut, Villigen PSI, 5232, Switzerland; 2 Synchrotron SOLEIL, L’Orme des Merisiers, 91190, Saint-Aubin, France; 3 Institute of Photonics, University of Eastern Finland, P. O. Box 111, FI-80101, Joensuu, Finland; 4 Center for Nanoscale Materials, Argonne National Laboratory, Lemont, IL 60439, United States

## Abstract

Multi-keV X-ray microscopy has been particularly successful in bridging the resolution gap between optical and electron microscopy. However, resolutions below 20 nm are still considered challenging, as high throughput direct imaging methods are limited by the availability of suitable optical elements. In order to bridge this gap, we present a new type of Fresnel zone plate lenses aimed at the sub-20 and the sub-10 nm resolution range. By extending the concept of double-sided zone plate stacking, we demonstrate the doubling of the effective line density and thus the resolution and provide large aperture, singlechip optical devices with 15 and 7 nm smallest zone widths. The detailed characterization of these lenses shows excellent optical properties with focal spots down to 7.8 nm. Beyond wave front characterization, the zone plates also excel in typical imaging scenarios, verifying their resolution close to their diffraction limited optical performance.

A longstanding dream of many scientists is a microscope offering the complementary capabilities of optical and electron microscopy while bridging the resolution gap between these two methods. An instrument with the ability to resolve chemical states and electronic structure at the molecular scale in three dimensions and within microns-thick, unsectioned specimens, would have a transformative impact on the life and materials sciences. Elucidation of the higher order packing of chromatin in metaphase chromosomes, and unfolding the coupling between intertwined lattice and spin order parameters in correlated electron materials are but two examples begging for this capability.

X-ray microscopy^1^ has been spectacularly successful for studying thick specimens with nanoscale resolution using photons in the hard X-ray (multi-keV) range. Coherent diffractive imaging methods like ptychography have reached spatial resolutions in two dimensions of 5 nm in the soft^2^ and 8 nm in the hard X-ray range^3^ and have also demonstrated 16 nm isotropic resolution in X-ray tomography^4^. These methods are advancing rapidly, however they only access X-rays elastically scattered by the sample, and numerical inversion of the measured diffraction data is necessary to obtain images of the sample structure. X-ray imaging with lenses – also referred to as real-space microscopy – offers additional contrast mechanisms such as X-ray fluorescence and directly yields sample images on its own, or by complementing other methods^5^. Reaching a resolution better than 10 nm with lens-based X-ray microscopy has not only been extremely challenging but was even considered to be impossible until relatively recently^6^,^7^,^8^. State of the art X-ray microscopes have demonstrated a resolution below 20 nm using Fresnel zone plate lenses in selected experiments^9^,^10^,^11^, but these experiments are still far from routine, partly due to lack of optical elements with sufficient resolution.

Diffractive X-ray optics, such as Fresnel zone plates^12^, are widely used as objective lenses for both scanning probe and full-field X-ray microscopy. These diffractive lenses can be described as circular gratings with decreasing periodicity towards their outer edge. The resolution of such elements is typically on the order of their smallest line width while the efficiency is mainly determined by their structure height. In the hard X-ray regime, optimal structure heights lie in the micron range even for the densest heavy metals. As the line width decreases, limitations in fabrication possibilities introduce severe restrictions on the structure quality. Conventional zone plate fabrication processes using e-beam writing break down in the sub-20 nm feature range^13^,^14^. While the patterning of smaller structures is still possible, as the smallest zone width approaches the single-digit nanometre range, the feasible aspect ratio – the ratio of structure height and width – and thus the reachable efficiency suffers greatly. Although zone plates operating in higher diffraction orders have been utilized for high resolution imaging in the soft X-ray regime^11^,^15^, their application to hard X-rays was limited by the low efficiency of this approach. Multilayer zone plates^16^ have successfully produced spot sizes below 10 nm, but their limited aperture size of a few microns, resulting in very short working distance has, so far prevented practical use. Combining two one-dimensional focusing elements with larger apertures, like Multilayer Laue lenses^17^,^18^,^19^ or K-B mirrors^20^ overcomes this limitation, however, the mounting and aligning two of these elements requires an extremely stable and drift-free setup^21^. Such arrangements require complicated alignment and have so far found limited applications in full-field microscopy besides proof-of concept experiments^22^,^23^.

The mechanical stacking of two separate zone plate chips^24^ is an established method to relax fabrication constraints of zone plate optics for the hard X-ray range. By placing two identical zone plates on top of one another’s optical near-field, they will act as a single zone plate with added transmission profile, *i.e.* double the structure height. Stacking can improve their efficiency by up to a factor of four beyond that of a single zone plate^25^,^26^,^27^. Recently, a new stacking method has been proposed^28^ that aims at increasing resolution instead of boosting efficiency by stacking two complementary zone plates. By patterning every even zone on one chip and every odd zone on the other, two complementary partial zone plates can be combined into a single zone plate as depicted in Fig. 1a. The combined zone plate exhibits half the periodicity and thus double the resolution as the actually fabricated individual partial zone plates. This approach is analogous to frequency doubling via interlacing in cathode-ray tube TVs and monitors, whereas the scanning electron beam draws only every even or odd line to double the refresh rate. Moreover, both partial zone plates can be fabricated using the zone doubling technique^13^,^29^. In this method, firstly a sparse resist template has to be fabricated by direct e-beam patterning (Fig. 2). Second, the template is coated with high density metals using atomic layer deposition. As the resist is transparent to hard X-rays, the conformal metal coverage on the sidewalls of the template will act as the de-facto zones of the zone plate. Since each line has two sidewalls, one resist line results in two zones of the zone plate. This can be further doubled by interlaced stacking, resulting in an effective frequency multiplication by a factor of four (Fig. 1b). In particular, this method also avoids the patterning of very narrow resist structures as required for conventional zone-doubling.

Therefore, frequency multiplication offers the potential for ultra-high resolution zone plate optics without sacrificing structure height and thus efficiency in the hard X-ray regime. Yet, stacking presently suffers from major limitations regarding alignment, stability and mechanical complexity^25^. As an alternative to mechanical stacking, we have shown that the every-day use can be greatly simplified by fabricating the two partial zone plates on both sides of the same support membrane^30^,^31^. This allows to substitute mechanical stacking with a single-chip solution (Fig. 1c) by exchanging the mechanical alignment of two chips with the alignment of the two sides during the patterning process.

In this article, we report on the fabrication and characterization of two sets of double-sided, line-doubled Fresnel zone plates with 100 microns aperture size. The individual sets of zone plates exhibit approximately 300 nm high iridium zones with 15 nm and 7 nm as smallest outer zone width. With the latter, we aim at providing sub-10 nm focusing capabilities with a large aperture, convenient optical element for ultra-high resolution hard X-ray microscopy. The zone plate parameters were chosen to avoid negative effects regarding the finite stacking distance^28^.

The individual partial zone plates were fabricated using a modified zone doubling technique^10^ to facilitate the alignment of the two sides. The alignment of the two sides is extremely critical for high resolution double-sided zone plates as it had to be performed within a few nanometres accuracy in our case^32^. Therefore the 100 nm thick silicon nitride support membranes were first pre-patterned with conductive layers from both sides and a set of gold alignment markers on the front side of the membrane. These markers could be accurately located using our electron beam writer’s alignment procedure even through the thin silicon nitride membrane. We patterned each side of the membrane separately by the direct writing of a HSQ resist template using electron beam lithography. In order to achieve the sufficient overlay accuracy, both the back and front side exposures had to be aligned on the very same set of four markers. After the patterning of both sides, the templates were conformally coated with iridium using atomic layer deposition.

## Results

### Wavefront characterization

**Figure 3:**
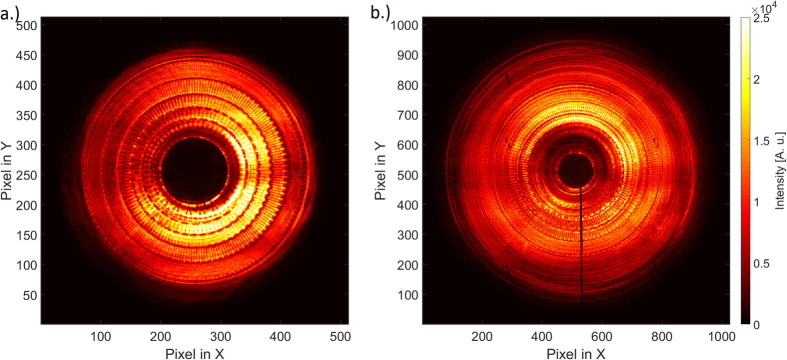
Diffraction cones of the selected zone plates with (**a**) 15 nm and (**b**) 7 nm smallest zone width at 9 keV. Apart from the illuminating beam profile, the diffraction cones exhibit a smooth efficiency distribution. The absence of Moire fringes confirms correct alignment between the two sides.

In order to qualify the fabricated zone plates and evaluate their optical properties in the X-ray range, we characterized selected double-sided zone plates with smooth efficiency distribution, seen on Fig. 3, at 9 keV photon energy using ptychography^33^,^34^ at the cSAXS beamline of the Swiss Light Source.

**Figure 4:**
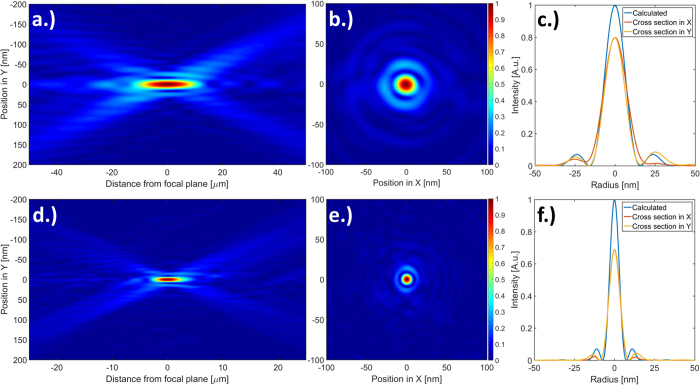
Beam propagation of the 15 nm and 7 nm interlaced zone plates measured using ptychography at 9 keV. (**a**) Wavefield amplitude, (**b**) focal plane amplitude and (**c**) focal spot intensity cross-sections of the highest efficiency 15 nm interlaced zone plate. (**d, e & f**) Corresponding plots for the characterized 7 nm zone plate. Both focal spots do not show evident signs of optical aberrations and exhibit a single focal spot with an approximately regular first Airy ring.

The reconstructed illuminations were back-propagated to the focal plane to recover the shape of the focal spot^3^,^35^,^36^. Figure 4 shows the wavefield amplitude along the optical axis and the intensity distribution in the focal plane. Both illuminations exhibit a single circular maximum and have no apparent aberrations. Misalignment between the two exposures on different sides would manifest as two focal spots or evident distortion of the first Airy ring. Thus the reconstructions in Fig. 4 confirm the accurate alignment of the two sides. The focal spot of the tested 7 nm zone plate displays a full width at half-maximum of 7.8 nm in the horizontal and 7.9 nm in the vertical direction. This slight ellipticity may be attributed to uneven illumination of the zone plate or a slight misalignment of a fraction of the outermost zone width.

As a quantitative evaluation of the optical quality, the Strehl ratio^37^,^38^,^39^ is a good indicator for small aberrations. The highest efficiency 15 nm zone plate reached a Strehl-ratio of 0.78, which is close to diffraction limited imaging quality of 0.8^40^. For the 7 nm zone plate, the sidelobes were noticeably stronger than for the 15 nm zone plates, which leads to a lower Strehl-ratio of 0.69. A root-mean-square phase error of *σ* = 0.095*λ* of the focused beam was calculated by back propagating the reconstructed beam to the plane of the lens^3^.

### Scanning and full-field transmission X-ray microscopy using interlaced zone plates

As ptychographic characterization predicts both zone plates to be of good optical quality, they should provide sufficient contrast for imaging close to their diffraction limit. In order to demonstrate the imaging performance of the ultra-high resolution zone plates, selected zone plates were tested in either scanning probe microscopy or full-field transmission microscopy measurements.

**Figure 5:**
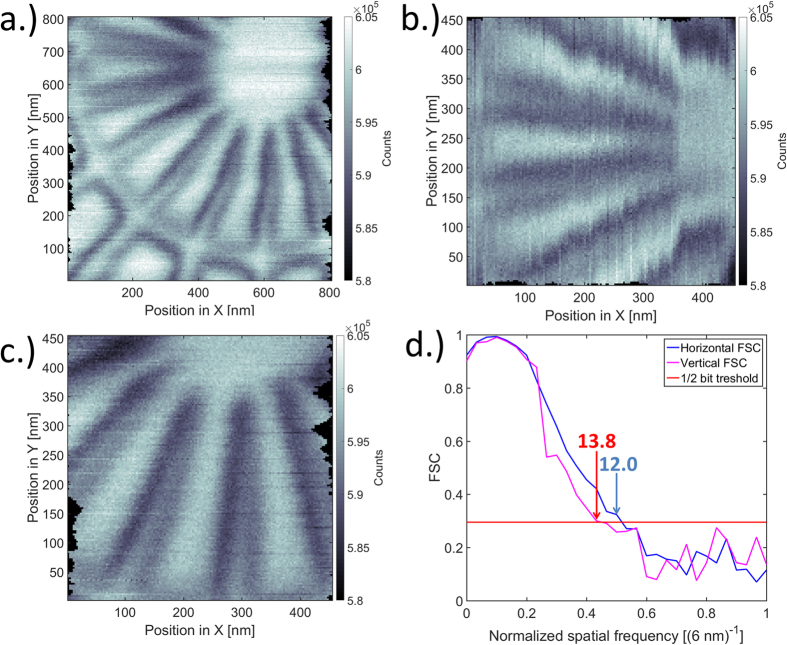
Drift-corrected STXM images of a Siemens star test pattern at 9 keV. A star test pattern with 30 nm external and 10 nm internal spoke size was recorded using a 7 nm Fresnel zone plate as objective. Image (**a**) shows a discrepancy in resolution between the fast and slow scanning axes. Therefore, the fast axis was selected perpendicular to the structures in (**b,c**). (**d**) FRC along the fast scanning direction from scans shown in (**b,c**) and the 1/2 bit threshold curve. Along the fast axis, the resolution is limited to ~12 nm presumably due to positioning accuracy, drifts and coherent flux.

The 7 nm zone plate was tested to focus a beam for scanning transmission X-ray microscopy (STXM). Using the same setup as for ptychography, the measurement was performed by continuously scanning a test object with feature sizes down to 10 nm, while recording the transmitted intensity. As seen on Fig. 5a, the obtained resolution was different along the fast and slow scanning axes. Figure 5b and c show images acquired with horizontal and vertical fast axes resulting in 12.0 nm horizontal and 13.8 nm vertical resolution respectively (Fig. 5d). It is important to note, that these values are attributed to technical limitations in the experimental setup, rather than to the zone plate performance. Using a dedicated STXM endstation with interferometric control and high acquisition speed, we expect the resolution to improve below 10 nm. The recorded images verify the result from the ptychographic reconstructions and demonstrate that the 7 nm zone plate is indeed in practice capable of image formation with resolution close to its expected limit. However, it also shows, that high-performance X-ray optics require instrumentation with sufficiently high stability and coherent flux to exploit their full potential in terms of resolution.

**Figure 6:**
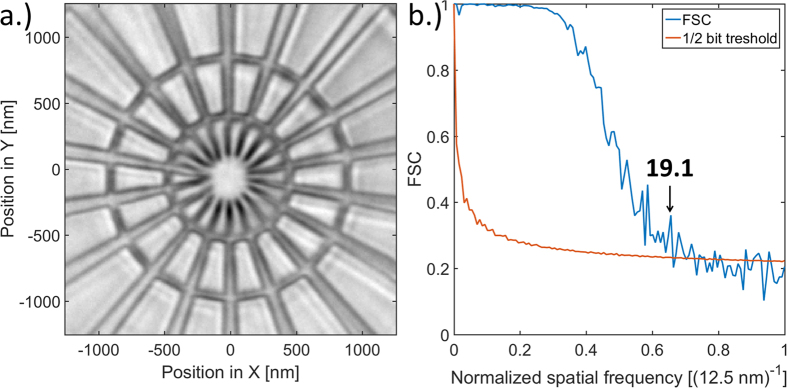
Full-field X-ray microscopy with a 15 nm Fresnel zone plate. (**a**) Full-field X-ray micrograph of a Siemens star test object using the highest efficiency 15 nm Fresnel zone plate as objective lens at 9 keV. The internal structure of the spokes is partly resolved, which supports the 19.1 nm resolution from FRC on (**b**).

The 15 nm zone plate was tested as an objective lens for full-field transmission X-ray microscopy. The inspected specimen was a similar test object as used for STXM. The acquired micrograph shown in Fig. 6 reveals some of the internal structures of the spokes. The image resolution is measured to be 19.1 nm using FRC, which almost meets the theoretical value of 18.3 nm for the aforementioned zone plate.

### Efficiency measurements

Besides imaging performance, efficiency is also an important property for zone plate optics working in the hard X-ray range. Therefore, the overall efficiency of the zone plates together with an efficiency map of the projected radiation cone was obtained by recording their first diffraction order using a 2D detector^41^ 7.3 m downstream of the focal point and comparing its intensity to the illuminating flux at 9 keV photon energy. From more than a dozen zone plates with 15 nm effective outer zone width, the measured efficiencies reached up to 0.92% (compared to the ideal calculated value of 1.9%), which is considerably higher than described in prior reports^13^. The 7 nm zone plates yielded efficiencies up to 0.52%, which is also remarkable when taking into account the increased volume effects at these structure sizes, as detailed by prior authors^28^.

### Efficiency modeling using the coupled wave theory

Prior calculations in the literature anticipated very low focusing efficiencies for interlaced zone plate stacks in the sub-10 nm regime^28^. The authors in ref. 28 imply that a correction to volume effects becomes unavoidable and a slight shift of the zones from their original position for the second zone plate of the stack is required to obtain reasonable efficiencies in agreement with the concept of intermediate field stacking^42^. In order to resolve the potential conflict between the prior work and the measured efficiency values, the diffraction efficiency of stacked periodic structures as used in the presented zone plates was simulated using the coupled wave theory^43^,^44^. Thus we created a realistic geometric model of stacked periodic structures, corresponding to the smallest zones of double-sided zone plates (Fig. 6a) to calculate their theoretical efficiency for our particular geometry including the aforementioned volume effects.

**Figure 7:**
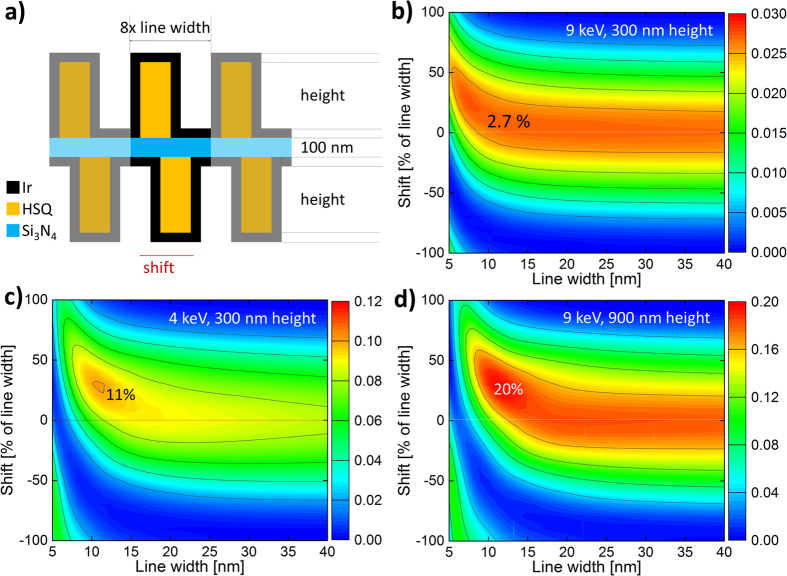
Efficiency calculations using coupled wave theory. (**a**) Schematic illustration of the simulated model for efficiency calculations. The grating consists of seven layers in the propagation direction, and the unit cell corresponds to eight times the iridium thickness. (**b**) Calculated first order diffraction efficiency of an interlaced grating as a function of the line width and the shift of the structures in units of line width, as schematically shown in (a), corresponding to the studied experimental setup at 9 keV and a structure height of 300 nm. (**c**,**d**) The same calculations for 4 keV and 300 nm structure height and 9 keV and 900 nm structure height. The benefits of higher maximum efficiencies with increased thickness or lower energies are diminished in the sub-10 nm regime.

Figure 7b shows the expected efficiency of the first diffraction order for a given smallest zone width, *i.e.* iridium thickness, as a function of the shift of the lower half of the grating versus the line width. The calculations were performed with 9 keV photon energy and a structure height of 300 nm on each side of the membrane, in accordance with our experimental conditions. Due to the different geometry with 300 nm instead of 900 nm structure height, the zone plate efficiency does not drop sharply for 7 nm zones even with unadjusted zone placement. The contour plot appears to be rather symmetric down to approximately 15 nm zone width. At smaller line widths, volume effects start to favour a slight shift of the zones, going up to 25% of the line width at 7 nm (1.75 nm shift). The simulations were repeated for a photon energy of 4 keV and a structure height of 900 nm. Both the lower energy or the higher structures provide higher efficiency, which manifests in higher maximum efficiency values (11% and 20%). However, the gain of efficiency using optically thicker structures or lower photon energies diminishes quickly. Below 10 nm zone width the need for a slight pattern shift becomes more pronounced. This indicates that the tested 300 nm structure height and 9 keV photon energy represent a good design compromise for interlaced zone plates.

## Discussion

In this work, we demonstrate the feasibility, realization and practical application of high resolution, frequency doubled hard X-ray Fresnel zone plate optics following the interlaced stacking scheme. By exchanging mechanical stacking with double sided patterning using aligned e-beam exposures, we provided interlaced optics with unprecedented resolution and large aperture sizes.

Ptychographic characterization of the selected lenses confirmed the expected beam size and quality. The zone plates with 7 nm smallest half-pitch have been shown to provide down to 7.8 nm spot FWHM, which is to our knowledge the highest resolution obtained with large aperture Fresnel zone plate optics. Moreover, the tested zone plates did not show signs of severe aberration and were of high optical quality even for the 7 nm smallest zone width. Besides ptychographic characterization, the tested optics also performed well in imaging experiments. Acquired full-field and STXM micrographs verified the expected performance, yet they were clearly limited by mechanical instabilities in the rest of the beamline instrumentation.

Using numerical methods, we have verified that – despite of prior calculations – frequency doubled zone plate stacks in the right geometry can indeed provide substantial efficiency even for spot sizes below 10 nm. In line with the calculations, the tested zone plates delivered focusing efficiencies of 0.92% for 15 nm and 0.52% for 7 nm smallest zone width. The efficiency can be substantially improved for future applications by additional process development to increase sidewall verticality and ensure the correct profile and fill factor of the HSQ template, as well as by fine-tuning the thickness of the deposited iridium layer. Yet drastic improvements with generally applicable zone plates are unlikely with this design due to volume effects below 10 nm feature sizes. These can be partially overcome by custom optics, where the zone placement is adjusted to provide the best performance for the particular setup with fixed illumination characteristics.

In summary, we have shown, that interlaced stacking is a viable method for frequency multiplication, enabling the fabrication of hard X-ray Fresnel zone plates with resolution reaching deep into the nanometre regime. We demonstrated the concept with the quantitative characterization of hard X-ray Fresnel zone plate optics down to 7.8 nm focal spot FWHM, while still maintaining a practically useful aperture size and efficiency. Moreover, our characterization went beyond simple proof of concept experiments, as we have also evaluated the performance of our optics in imaging experiments that verified the results of the ptychographic measurements. However, the latter experiments also demonstrated that high-performance X-ray optics require instrumentation with sufficiently high stability and coherent flux to exploit their full potential in terms of resolution.

## Methods

### Zone plate fabrication

The fabrication scheme employed for the interlaced zone plates is closely related to the fabrication of double-sided line-doubled zone plates described in earlier works^30^. The zone plates were prepared on 100 nm thick and 2 × 2 *mm*^2^ silicon nitride membrane windows on 6 × 6 *mm*^2^ silicon frames. The substrates were coated with a Cr/Au/Cr conduction layer on both sides and were pre-patterned with gold alignment markers using electroplating^45^. The two sides of the membrane were patterned subsequently, starting with its back side. The processed side of the membranes were spin-coated with 300 nm hydrogen silsesquioxane (HSQ, FOX16 Dow Corning) electron beam resist. The partial zone plate patterns were directly exposed into the resist using a Vistec EBPG 5000 + ES electron beam writer with 100 keV acceleration energy. The two individual exposures on the front- and backside were aligned precisely with respect to each other using exactly the same set of four gold markers. The markers could be located even through the thin membrane with an accuracy of a few nanometres. The patterns on each side were developed in a *NaOH*-based salty developer (A351, Microposit) for 10 minutes and immediately dried by supercritical drying after the patterning of each side. After the production of the HSQ support patterns on both sides of the membranes, the nanostructures were coated with 15 nm or 7 nm thick iridium films using atomic layer deposition^46^.

### Efficiency measurements

The preliminary characterization was performed by illuminating the zone plate through a pinhole of equal size as the zone plate and selecting the first diffraction order using a pinhole with 10 *μ*m diameter in the focal plane. The defocused diffraction cones were recorded 7.3 m downstream of the focus on a PILATUS 2 M single-photon counting pixel detector^41^. The total counts recorded on the detector were normalized to the photon flux by direct, photodiode based efficiency measurements^30^.

### Ptychographic characterization

The ptychographic characterization was performed on a Siemens-star test object with 10 nm smallest feature size, mounted slightly downstream of the focus to obtain a 1.5 *μ*m wide illumination cone on the test sample. For each reconstruction, a 5 × 5 *μm*^2^ area was sampled in 200 nm steps in a Fermat spiral scanning pattern^47^. The scan was repeated in two detector positions to account for the missing spatial frequencies due to detector gaps and the reconstruction of the two scans was performed simultaneously by sharing between them a common illumination^48^. Since the diffraction cones of the Fresnel zone plates with high numerical apertures are extended over many pixels on the detector, the number of counts in the individual pixels was comparably low. In order to adequately account for the Poisson distribution of noise, the reconstructions were performed using the maximum likelihood method^49^.

### Scanning probe microscopy

After ptychographic characterization, a selected 7 nm zone plate was tested as an objective lens for scanning transmission X-ray microscopy in the same setup as for ptychography. The focal plane of the lens was located by numerical back propagation of the wavefield measured from ptychography and its position was refined by STXM scans at different positions along the optical axis. The measurements were performed by continuously scanning the test object while acquiring the transmitted intensity on the fly using a PILATUS 2 M detector^41^ at an acquisition rate of 7 frames per second. The recorded lines were corrected for fluctuations of the illuminating intensity due to continuous top-up mode of the synchrotron and aligned to each other using the autocorrelation of subsequent lines. As the obtained resolution was clearly different in the fast and slow directions, the horizontal and vertical resolutions were evaluated separately by changing the scan fast axis and moving to different sample positions with suitably oriented structures. Because the size of studied sample features and the scan step size of 3 nm are very close to the image resolution, it is difficult to visually asses the latter. As a quantitative value, the image resolution was calculated using Fourier Ring Correlation (FRC)^50^. To avoid the influence of line-to-line changes, which artificially increase the correlation, a one dimensional version of the FRC was calculated. To reduce the negative effect on the SNR of the noise from empty regions we computed the FSC curves from 360 × 360 nm^2^ subregions of Fig. 5b,c using slow axis lines between 33 and 390 nm.

Each line of the scans was subdivided into an even and odd set of points, each line of these subsets was apodized at the edges to reduce edge effects, Fourier transformed and the correlation was calculated jointly from all 120 lines and the resolution estimated using the half-bit threshold criteria^50^, yielding values down to 12.0 nm resolution, that fits well with visual evaluation. The results were cross-checked with power spectral analysis (not shown here) that verified the presence of signal at the corresponding spatial frequencies.

### Full-field microscopy

The highest efficiency zone plate with 15 nm smallest zone width was tested as the objective of the full-field microscope at the Hard X-ray Nanoprobe beamline at the Center for Nanoscale Materials at the Advanced Photon Source^51^ using 9 keV photon energy. A similar Siemens star test sample was illuminated by a capillary condenser^52^ with a numerical aperture roughly equivalent to a zone plate condenser of 25 nm smallest zone width. The objective was placed approximately one focal length downstream of the sample that was followed by an evacuated flight tube and a scintillator screen that was projected onto a CCD detector (Princeton PIXIS-XF). This corresponds to an effective pixel size of 12.5 nm on the image. The final image was produced by aligning and averaging 80 images after flat-field correction (Fig. 6) by iteratively maximizing their autocorrelation with the final image. The resolution was calculated using two dimensional FRC between the average of two subsets of 40 images.

We were unable to test the performance of zone plates with 7 nm smallest zone width. As line-doubled zone plates have reduced efficiency near their centre, they rely on a well-matched condenser NA to spread the low spatial frequencies across the whole zone plate. With the heavily disproportional condenser and objective numerical aperture as was available for our tests, the low spatial frequencies were lost and the microscope was limited to dark-field contrast. The latter effect would disappear with a better matched, higher numerical aperture condenser.

### Calculation of the Strehl ratio and wavefront error

As a single value benchmark, the Strehl ratio compares the relative peak intensity of the focal spot compared to its theoretical value. It is a good indicator of the relative intensity of the peak versus the surrounding sidelobes. Optical elements are generally considered as diffraction limited with Strehl ratios above 0.8. For accurate comparison, the illumination reconstructed using ptychography was propagated back to the focal plane of the zone plate to obtain the reconstructed focal spot. The total intensity within 1 *μm* around the focal spot was normalized with the expected total intensity from a 100 *μm* annular aperture with a 35 *μm* central stop and the measured peak intensity was divided by the theoretical one to produce the Strehl ratio. The wavefront error was determined by propagating the reconstructed probe to the focus, where the quadratic phase term of the lens was removed, and subsequently was propagating it to the far-field. The phase error was calculated only from areas contributing at least 1 photon to the average diffraction pattern and was weighted by the local efficiency.

### Coupled wave theory analysis

The coupled wave theory analysis is an established numerical method for the simulation of volume effects in diffraction gratings in the X-ray range^44^. In order to achieve reliable results, we created a realistic grating model of the smallest zones of our double-sided interlaced line-doubled zone plates. The model includes the HSQ template, the 100 nm thick silicon nitride membrane, the iridium zones on the sidewalls, and the iridium coating on the membrane and on top of the HSQ lines (Fig. 7a). The resulting model consists of seven layers along the direction of propagation, three on each side of the membrane plus the support membrane itself. Each unit cell comprises eight times the coating thickness, which corresponds to the effective line width. In agreement with the quadrupling of the resolution, each unit cell consists of four iridium lines, resulting in the calculated values for the fourth diffraction mode of the grating being the first diffraction order of the stacked zone plate.

## Additional Information

**How to cite this article**: Mohacsi, I. *et al*. Interlaced zone plate optics for hard X-ray imaging in the 10 nm range. *Sci. Rep.* **7**, 43624; doi: 10.1038/srep43624 (2017).

**Publisher's note:** Springer Nature remains neutral with regard to jurisdictional claims in published maps and institutional affiliations.

## Figures and Tables

**Figure 1:**
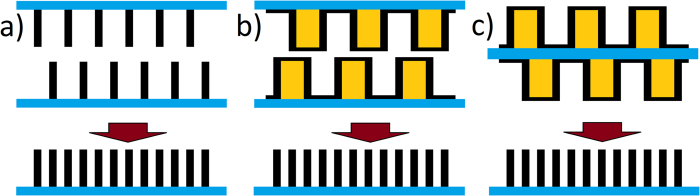
Interlaced stacking of Fresnel zone plates. (**a**) The interlaced stacking of zone plates places two complementary partial zone plates in each other’s optical near-field. The structures will add up in transmission leading to half the pitch and two times better resolution compared to the actually patterned sparse nanostructures. (**b**) The same arrangement with partial zone plates made by line-doubling. Only the sparse and thick resist structures (yellow) need to be prepared by lithography, while the actual zones (black) are deposited using atomic layer deposition. This effectively quadruples the resolution compared to the actually patterned template. (**c**) Interlaced stacking realized with double-sided zone plates on two sides on the same membrane.

**Figure 2:**
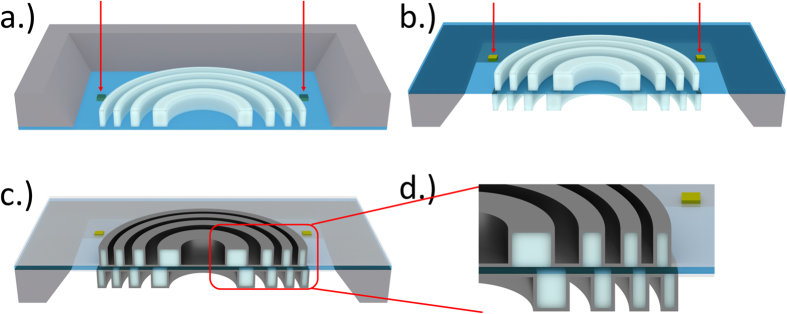
Schematic fabrication process for double-sided interlaced zone plates using the zone-doubling technique. (**a**,**b**) The back and front side of the membrane is patterned with a resist template in two separate exposures, aligned on the same set of markers. (**c**) The template is conformally coated with an iridium layer. (**d**) A closer look shows the interlaced placement of the iridium sidewalls.
